# Accuracy when inferential statistics are used as measurement tools

**DOI:** 10.1186/s13104-016-2045-z

**Published:** 2016-04-26

**Authors:** Michael T. Bradley, Andrew Brand

**Affiliations:** Department of Psychology, University of New Brunswick, 100 Tucker Park Road, PO Box 5050, Saint John, NB E2L 4L5 Canada; NWORTH Bangor Clinical Trials Unit, Institute of Medical & Social Care Research, Bangor University, Bangor, UK

**Keywords:** Accuracy, Measurement, Neyman and Pearson, Probabilities, Type 1 errors, Type 2 errors

## Abstract

**Background:**

Inferential statistical tests that approximate measurement are called acceptance procedures. The procedure includes type 1 error, falsely rejecting the null hypothesis, and type 2 error, failing to reject the null hypothesis when the alternative should be supported. This approach involves repeated sampling from a distribution with established parameters such that the probabilities of these errors can be ascertained. With low error probabilities the procedure has the potential to approximate measurement. How close this procedure approximates measurement was examined.

**Findings:**

A Monte Carlo procedure set the type 1 error at p = 0.05 and the type 2 error at either p = 0.20 or p = 0.10 for effect size values of d = 0.2, 0.5, and 0.8. The resultant values are approximately 15 and 6.25 % larger than the effect sizes entered into the analysis depending on a type 2 error rate of p < 0.20, or p < 0.10 respectively.

**Conclusions:**

Acceptance procedures approximate values wherein a decision could be made. In a health district a deviation at a particular level could signal a change in health. The approximations could be reasonable in some circumstances, but if more accurate measures are desired a deviation could be reduced by the percentage appropriate for the power. The tradeoff for such a procedure is an increase in type 1 error rate and a decrease in type 2 errors.

## Findings

### Background

A hallmark of science is measurement, and Neyman and Pearson [1] contributed a way of approximating measurement with a probability model derived from inferential statistical situations. They described type 1 error rates with the known parameters of a distribution such that a value that deviated from the mean by a specific amount or more could be considered a true deviation with only a 5 % chance of being wrong. With a type 2 error rate they could specify the chance of failing to find this value as only 10 to 20 % of the time. In doing this they developed a paradigm such that it might be reasonable to calculate a measurement value following the Neyman and Pearson [1] method under the proper conditions. In fact, Wald [2] used inferential tests to make decisions in manufacturing processes.

To ascertain the value of using inferential statistics to make decisions a variety of effect size values (d = 0.2, 0.5, 0.8) under the sample size conditions appropriate to a type 1 error of p < 0.05 and a type 2 error rates of 0.2 or 0.1 were tested in a Monte Carlo deign to see how close the resulting effect sizes are to the initial value. In effect this is a test of the accuracy of Neyman and Pearson’s [1] approach.

## Methods

A control distribution with a normal distribution of 1,000,000 values and a mean of 10 and a standard deviation of 2 was generated. Three experimental distributions were created so that the difference between the control distribution and an experimental distribution corresponded to one of Cohen’s [3] definitions of small (d = 0.20), medium (d = 0.50) and large (d = 0.80) effect sizes. The means for the experimental distributions for the small, medium and large effect sizes were 10.40, 11.00, and 11.60, respectively and the standard deviations of the experimental distributions were the same as the control distribution (i.e., 2). To determine the sample size per group required to achieve 80 % statistical power for each of the three effect sizes, three separate power analyses was conducted. The results from these power analyses showed that sample size per group required to achieve 80 % statistical power to detect the small, medium and large effect size was 393, 64 and 26 respectively. Then for each of the three effect sizes, 100,000 experiments were simulated using the sample size obtained from the power analysis and a two-tailed between-subjects t test was conducted for each simulated experiments. The effect size estimates with error rates of 0.05 were derived by calculating and collating the effect size estimates for only the simulated experiments where the p value from the between-subjects t test was statistically significant (i.e., p < 0.05). This procedure was then repeated using a sample size per group of 527, 86, 34 that achieved 90 % statistical power to detect the small, medium and large effect respectively.

## Results

With error rates of 0.05 for type 1 and type 2 of 0.20 and 0.01, (80 and 90 % power) the Monte Carlo analysis showed effect sizes estimates as follows:

80 % Power:

- True effect = 0.2, estimate = 0.23, 15 % larger
- True effect = 0.5, estimate = 0.56, 12 % larger
- True effect = 0.8, estimate = 0.91, 13.75 larger

90 % Power:

- True effect = 0.2, estimate = 0.21, 5 % larger
- True effect = 0.5, estimate = 0.53, 6 % larger
- True effect = 0.8, estimate = 0.85, 6.25 larger

**Fig. 1:**
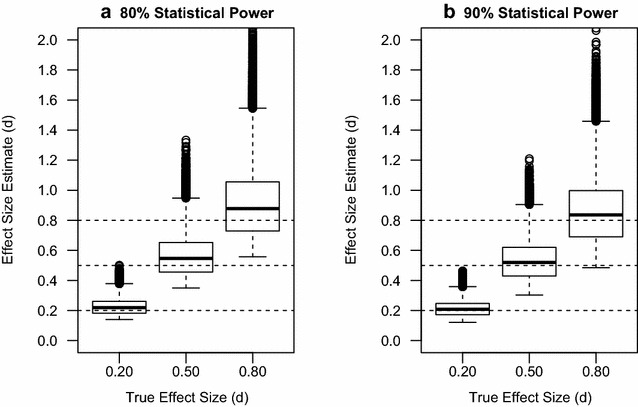
Effect size estimates when Statistical Power is **a** 80 % and type 1 error rate = 0.05 **b** 90 % and type 1 error rate = 0.05

Figure 1 illustrates the results.

## Discussion

The implications of this demonstration are important. An analyst focused on the p < 0.05 level for both of these power levels would find the estimated effect size is larger than the actual effect size in all cases. The maximum exaggeration is 15 % with a type 2 error rate of 0.20. A decrease in the type 2 error rate or its reciprocal an increase in power to 90 % brings the maximum measurement error down to 6.25 %. Thus making decisions based on this model could be quite reasonable if statisticians are dealing with a well described population.

It must be remembered, however, that the conditions of repeated sampling from a distribution so well described as to yield specified type 1 and type 2 errors is specific to situations involving, for example, a highly developed technology. When the distribution changes and error rates vary such that sample size and therefore power varies this model would not pertain. Fisher [4, p 80] was conscious of what he called “acceptance procedures”, and stated correctly they were not appropriate for exploratory scientific research. On the other hand, a medical monitoring system within a known population for a district could use this Neyman and Pearson approach for assessing changes in health. Measurement accuracy could be increased by reducing the effect size that could be a “signal deviation” by 5 or 6 % with 90 % power or 12 to 15 % with 80 % power. Of course, there is a value judgement since such a procedure increases the type 1 error rate and decreases the type 2 error rate.

## Conclusions

If an analyst follows the recommendations of Neyman and Pearson [1] they will approximate measurement but only with a known distribution and accepting that any effect size calculated from the approximation is inherently somewhat exaggerated.
